# IS*26*-Mediated Genetic Rearrangements in *Salmonella* Genomic Island 1 of *Proteus mirabilis*

**DOI:** 10.3389/fmicb.2019.02245

**Published:** 2019-09-24

**Authors:** Xue-Chun Wang, Chang-Wei Lei, Zhuang-Zhuang Kang, Yu Zhang, Hong-Ning Wang

**Affiliations:** Animal Disease Prevention and Food Safety Key Laboratory of Sichuan Province, Key Laboratory of Bio-Resource and Eco-Environment of Ministry of Education, College of Life Sciences, Sichuan University, Chengdu, China

**Keywords:** *Salmonella*, *Proteus*, genomic island, SGI1, multidrug resistance, IS*26*

## Abstract

*Salmonella* genomic island 1 (SGI1) is an integrative mobilizable element integrated into the chromosome of bacteria, which plays an important role in the dissemination of antimicrobial resistance genes. Lots of SGI1 variants are found mainly in *Salmonella enterica* and *Proteus mirabilis*. In this study, a total of 157 *S. enterica* and 132 *P. mirabilis* strains were collected from food-producing animals in Sichuan Province of China between December 2016 and November 2017. Detection of the SGI1 integrase gene showed that three *S. enterica* and five *P. mirabilis* strains were positive for SGI1, which displayed different multidrug resistance profiles. Five different SGI1 variants, including two novel variants (SGI1-PmBC1123 and SGI1-PmSC1111), were characterized by whole genome sequencing and PCR linkage. In two novel SGI1 variants, IS*26*-mediated rearrangements resulted in large sequence inversions of the MDR regions extending outside the SGI1 backbone. The *sul3*-type III class 1 integron (5′CS-*sat*-*psp*-*aadA2*-*cmlA1*-*aadA1*-*qacH*-IS440-*sul3*) and gene cassettes *aac(6*′*)-Ib-cr-bla*_OXA–__1_-*catB3*-*arr-3* are found in SGI1-PmSC1111. Mobilization experiments indicated that three known variants were conjugally mobilized in *trans* to *Escherichia coli* with the help of a conjugative IncC plasmid. However, the two novel variants seemed to lose the mobilization, which might result from the sequence inversion of partial SGI1 backbone. The identification of the two novel SGI1 variants in this study suggested that IS*26*-mediated rearrangements promote the diversity of SGI1.

## Introduction

Genomic islands (GIs), such as integrative and conjugative elements (ICEs) and integrative mobilizable elements (IMEs), are distinct regions integrated into the chromosome of bacteria and acquired via horizontal transfer (Bellanger et al., 2014; Partridge et al., 2018). GIs often contain various genes endowing theirs hosts with new traits, like antimicrobial resistance and virulence that enhance bacterial adaptation to environment (Bellanger et al., 2014). *Salmonella* genomic island 1 (SGI1) is an IME initially identified in the multidrug resistance (MDR) *Salmonella* Typhimurium DT104 clone (Boyd et al., 2001). SGI1 (42.4 kb) is comprised of a backbone containing 28 ORFs (S001-S027 and S044) and a 13 kb MDR region that consists of a complex In4-type class 1 integron named In104 (Boyd et al., 2001; Levings et al., 2005). It can form extrachromosomal circular form and is specifically mobilized in *trans* by conjugative IncA/C plasmids (Doublet et al., 2005; Douard et al., 2010). In recent years, the mobilization mechanism of SGI1 with the help of IncA/C plasmids has been revealed in some studies and many aspects have been explored including the basic transfer elements (Carraro et al., 2014; Kiss et al., 2015, 2019; Siebor et al., 2016). SGI1 was reported in *Proteus mirabilis* in 2007 (Ahmed et al., 2007), and recently found in *Morganella morganii*, *Providencia stuartii* and *Escherichia coli* (Schultz et al., 2017a; Cummins et al., 2019; Soliman et al., 2019), indicating that SGI1 has a broad host bacterial range and has the potential to spread among enterobacteria.

Since the first report of SGI1 in *S.* Typhimurium DT104, many different SGI1 variants have been described (Hall, 2010; Bi et al., 2011; Siebor and Neuwirth, 2011, 2013; Lei et al., 2014; Lei et al., 2015; Qin et al., 2015; Schultz et al., 2015; Bie et al., 2018; de Curraize et al., 2018), most of which result from various insertion sequences, homologous recombinations, transpositions, and loss or exchange of gene cassettes within the MDR region (Hall, 2010). A deletion of 2,780 bp in size from part of ORFs S005 to S009 that is replaced by IS*Vch4* is found in some variants like SGI1-K (Doublet et al., 2008). Besides, several SGI1 related islands (SGI1, PGI1, AGI1, PGI2 and GI*Pmi*1) have been reported (Levings et al., 2008; Siebor and Neuwirth, 2014; Girlich et al., 2015; Hamidian et al., 2015; Lei et al., 2018b; Siebor et al., 2018, 2019), all of which incorporate into chromosomes at a specific location (3′ end of the *trmE* gene) as SGI1. SGI1 and related islands often harbor various antimicrobial resistance genes including carbapenems resistance gene *bla*_NDM–__1_ (Girlich et al., 2015), extended-spectrum β-lactamase (ESBL) genes *bla*_VEB–__6_ and *bla*_CTX–M–__15_ (Siebor and Neuwirth, 2011; de Curraize et al., 2018), and fluoroquinolones resistance genes *qnrA1* and *qnrB2* (Siebor and Neuwirth, 2011; Lei et al., 2014), indicating SGI1 and related islands are important vehicles for clinically important resistance genes. Recently, many variants of SGI1 and SGI1-related islands have been characterized in *P. mirabilis*, which are summarized in Supplementary Table S1. These variants were found in *P. mirabilis* isolates from food, human, poultry, swine and other animals such as dog and horse.

In the present study, we characterized the SGI/PGI genomic islands in *Salmonella enterica* and *P. mirabilis* of food-producing animal origin in Sichuan Province of China and described two novel SGI1 variants in *P. mirabilis*.

## Materials and Methods

### Bacterial Strains and Detection of SGI1 and Relative Islands

A total of 157 *S. enterica* strains (61 from swine and 96 from chicken) and 132 *P. mirabilis* strains (74 from swine and 58 from chicken) isolated from diseased tissues or anal swabs of animals among 30 poultry and 30 swine farms in Sichuan Province between December 2016 and November 2017. All isolates were identified using an automated system (BD Diagnostic Systems, Sparks, MD, United States). The presence of SGI/PGI/AGI/GI*Pmi*1 was screened by PCR targeting the integrase gene (the primers used to detection are listed in Supplementary Table S2) (Schultz et al., 2017b). Positive PCR products were sent to Chengdu Qingke Biological Engineering Technology & Services Co., Ltd., and sequenced by ABI 3730xl DNA Sequencer (Applied Biosystems, United States).

### Antimicrobial Susceptibility Testing

Antimicrobial susceptibility testing for strains positive for SGI1/PGI1 was determined by the disk diffusion method according to CLSI guidelines. Antimicrobial agents included ampicillin (AMP), amoxicillin-clavulanic acid (AMC), cefoxitin (FOX), cefotaxime (CTX), ceftriaxone (CRO), chloramphenicol (CHL), florfenicol (FFC), nalidixic acid (NAL), ciprofloxacin (CIP), streptomycin (STR), spectinomycin (SPT), apramycin (APR), doxycycline (DOX), trimethoprim (TMP), sulfizoxazole (SUL), trimethoprim-sulfamethoxazole (SXT) and polymyxin B (PB). *E. coli* ATCC25922 was used as a quality control strain.

### Whole Genome Sequencing and Analysis

All SGI/PGI-positive strains were sequenced using Illumina HiSeq platform (400-bp paired-end reads with about 200-fold average coverage). The draft genomes were assembled using software SPAdes_3.12.0. The gaps among contigs that carried SGI1 fragments were filled in by PCR linkage. Because the complete genetic structures of SGI1 in strains PmBC1123 and PmSC1111 could not be assembled by PCR linkage, whole genomes of those two strains were further sequenced using PacBio RS II sequencing instrument (100-fold average read depth). The chromosomes were assembled into one scaffold using software SMRT portal v.3.2.0. The MDR regions were confirmed by PCR linkage between regions belonging to non-repeated genetic elements. Multi-locus sequence type of *S. enterica* and acquired antimicrobial resistance genes were identified by MLST 2.0^1^ and ResFinder 3.1^2^, respectively. The complete nucleotide sequences of SGI1 variants were analyzed using the BLAST programs^3^. SNPs from genomes of the strains positive for SGI/PGI were called and a phylogeny based on the concatenated alignment of the high quality SNPs was inferred using CSI Phylogeny 1.4^4^ with parameters as defaults.

### Mobilization Assays of SGI1

Many SGI1 variants can form the circular extrachromosomal forms that are conjugally mobilized *in trans* to other bacteria with the help of the conjugative IncA/C plasmid (Hall, 2010). The circular extrachromosomal forms of SGI1 variants were detected through two rounds of PCR amplification using primers listed in Supplementary Table S2. Mobilization assays were carried out as previously described (Siebor et al., 2016), using *E. coli* C600 harboring an IncC plasmid pR55 as recipient strain. Transconjugants were selected on *Shigella* and *Salmonella* agar plates containing 300 mg/L rifampicin and trimethoprim (30 mg/L)/streptomycin (30 mg/L). The transfer frequency of SGI1was determined by dividing the number of *E. coli* SGI1 transconjugants by the number of *P. mirabilis* or *S. enterica* donor cells (Douard et al., 2010). The transconjugants were further examined for the presence of SGI1 integrase gene and the location of SGI1 in *E. coli* with primers listed in Supplementary Table S2.

### Nucleotide Sequence Accession Numbers

The complete nucleotide sequences of two novel SGI1 variants and the genomes of *P. mirabilis* strains PmSC1111 and PmBC1123 were submitted to GenBank and assigned accession numbers MH998664 (SGI1-PmBC1123), MH998665 (SGI1-PmSC1111), CP034091 (PmBC1123) and CP034090 (PmSC1111), respectively. The Whole Genome Shotgun projects have been deposited at DDBJ/ENA/GenBank under the accession RQSE00000000 (SCMYP1), RQJQ00000000 (SC10), RQSD00000000 (SC968), RQSF00000000 (PmBC55), RQSG00000000 (PmSN55) and RQSH00000000 (PmDJ107).

## Results and Discussion

### Prevalence of SGI/PGI in *S. enterica* and *P. mirabili*s

Detection and sequence analysis of SGI/PGI/AGI/GI*Pmi*1 integrase gene showed that 3 *S. enterica* and 5 *P. mirabilis* strains were positive for SGI1. Antimicrobial susceptibility testing indicated those 8 strains displayed different MDR profiles (Table 1) (*P. mirabili*s is intrinsically resistant to doxycycline and polymyxin B). *S.* Albany strain SCMYP1 of swine origin exhibited resistance to the third generation cephalosporins and polymyxin B. Two *P. mirabilis* strains (PmSN55 and PmDJ107) of chicken origin were resistance to amoxicillin-clavulanic acid, cefoxitin and the third generation cephalosporins. Three SGI1-containg *S. enterica* strains belonged to different STs (Table 1). A total of 28,848 SNPs were called and phylogenetic analysis showed the five *P. mirabilis* strains were not clonally related.

**TABLE 1:** SGI1-containing *S. enterica* and *P. mirabilis* isolates characterized in this study.

**Strain**	**Species**	**Source**	**Antimicrobial resistance profile^a^**	**Sequence type**	**SGI1**	
					**Variant**	**Size (bp)**
SCMYP1	*S*. Albany	Swine	AMP, CTX, CRO, CHL, FFC, NAL, STR, SPT, DOX, TMP, SUL, SXT, PB	ST292	SGI1-F	42,647
SC10	*S*. Infantis	Chicken	AMP, CHL, FFC, DOX, TMP, SUL, SXT	ST32	SGI1-F	42,647
SC968	*S*. Derby	Chicken	CHL, FFC, STR, SPT, DOX, TMP, SUL, SXT	ST40	SGI1-I	42,777
PmBC55	*P. mirabilis*	Swine	CHL, FFC, STR, SPT, APR, TMP, SUL, SXT	–	SGI1-W	33,909
PmBC1123	*P. mirabilis*	Swine	AMP, CHL, FFC, NAL, CIP, STR, SPT, APR, TMP, SUL, SXT	–	SGI1-PmBC1123	58,069
PmSC1111	*P. mirabilis*	Swine	AMP, CHL, FFC, NAL, CIP, STR, SPT, APR, TMP, SUL, SXT	–	SGI1-PmSC1111	82,352
PmSN55	*P. mirabilis*	Chicken	AMP, AMC, FOX, CTX, CRO, CHL, FFC, NAL, CIP, STR, SPT, APR, TMP, SUL, SXT	–	SGI1-W	33,909
PmDJ107	*P. mirabilis*	Chicken	AMP, AMC, FOX, CTX, CRO, CHL, FFC, NAL, STR, SPT, APR, TMP, SUL, SXT	–	SGI1-W	33,909

*^*a*^*Proteus mirabilis* is intrinsically resistant to doxycycline and polymyxin B.*

### Characterization of SGI1 Variants

**FIGURE 1:**
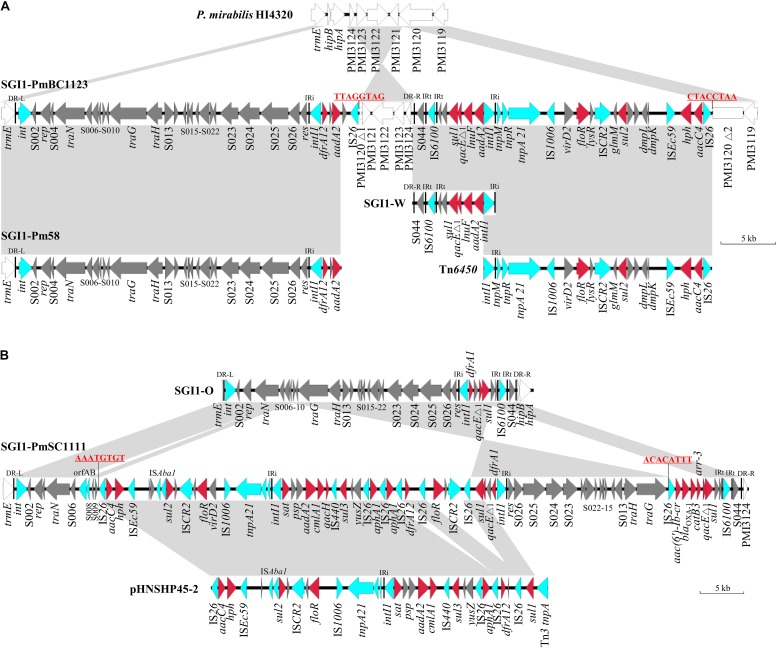
Genetic structures of SGI1-PmBC1123 **(A)** and SGI1-PmSC1111 **(B)**. Structures are drawn to scale from GenBank accession numbers MH998664 (SGI1-PmBC1123), MH998665 (SGI1-PmSC1111), AM942759 (*P. mirabilis* HI4320), KP662516 (SGI1-Pm58), MF805806 (Tn*6450*), KJ186151 (SGI1-W), KJ186150 (SGI1-O) and KU341381 (pHNSHP45-2). Genes and ORFs are shown as arrows, and their orientations of transcription are indicated by the arrowheads. Shared regions with above 99% identity are indicated by shading. Resistance genes are in red and transposase or integrase genes are in blue. DR-L and DR-R represent the 18-bp direct repeats at the ends of SGI1. IRi and IRt represent the inverted repeats defining the left and right ends of class 1 integron, respectively.

Five different SGI1 variants were identified in the eight strains through whole genome sequencing and PCR linkage. *S*. Albany strain SCMYP1 (ST292) and *S*. Infantis strain SC10 (ST32) harbors SGI1 variant SGI1-F that carries *dfrA1*-*orfC* and *bla*_CARB–__2_ (ampicillin) gene cassettes, as well as resistance genes *sul1* (sulfonamides), *tetA(G)* (tetracycline) and *floR* (chloramphenicol and florfenicol). *S*. Derby strain SC968 (ST40) harbors SGI1 variant SGI1-I that carries gene cassettes *aadA2* (streptomycin and spectinomycin) and *dfrA1*-*orfC*. Three *P. mirabilis* strains harbor SGI1 variant SGI1-W carrying *aadA2*-*lnuF* gene cassettes. SGI1-W was firstly detected in *P. mirabilis* of poultry origin in China (Lei et al., 2014), and then found in *P. mirabilis* and *P. stuartii* in Egypt (Soliman et al., 2017, 2019), suggesting this variant of SGI1 had the potential to spread among enterobacterial strains worldwide. It is interesting that two novel SGI1 variants, SGI1-PmBC1123 and SGI1-PmSC1111 (Figure 1), are characterized in this study for the first time.

SGI1-PmBC1123 is 58,069 bp in size (corresponding to bases 1,348-32,501 and 37,667-64,581 in accession no. MH998664) that carries gene cassettes *dfrA12*-*orfF*-*aadA2* and *aadA2*-*lnuF* found in SGI1-Z (Qin et al., 2015) and SGI1-W (Lei et al., 2014), respectively. The MDR region of SGI1-PmBC1123 is divided into two parts separated by a chromosomal DNA fragment of 5,148 bp (truncated PMI3120 and PMI3121-PMI3124). We hypothesize that an initial single IS*26* transposition event occurred in the chromosome in locus PMI3120 and has subsequently generated an inversion between two IS*26* elements in opposite orientation, resulting in the right end of SGI1-PmBC1123 and this 5,148 bp chromosomal DNA fragment being in inverse orientation. The similar phenomenon is also found in PGI1 (Siebor and Neuwirth, 2014). The 20.74 kb region in SGI1-PmBC1123 (corresponding to bases 43,839-64,581 in accession no. MH998664) that carries *floR*, *sul2* (sulfonamides), *hph* (hygromycin) and *aacC4* (apramycin) shows 99.9% nucleotide identity to the corresponding region of Tn*6450* (Chen et al., 2018), indicating they might have a common origin.

SGI1-PmSC1111 is 82,352 bp in size (corresponding to bases 1,348-83,699 in accession no. MH998665). A 1,258 bp insertion sequence, encoding transposase OrfAB subunits A and B, was inserted into the backbone gene S007 and flanked by 3-bp target site duplication (AAG). This IS element shows 98.6% nucleotide identity to IS*alg* firstly described in *Vibrio cholerae* serogroup O103 (Sozhamannan et al., 1999), and 86% nucleotide identity to IS*Vch4* in some SGI1 variants like SGI1-K (Doublet et al., 2008). An IS*26*-mediated recombination event occurred in S010, which caused the middle region of SGI1-PmSC1111 (corresponding to bases 10,880–75,577 in accession no. MH998665) being in inverse orientation and flanked by 8-bp inverted repeats (AAATGTGT) (Figure 1B). The downstream region of the 3′-partial of S010 (corresponding to bases 10,880–54,663 in accession no. MH998665) shows 99.9% nucleotide identity to the corresponding region of pHNSHP45-2 (accession no. KU341381) with the addition of two regions, *aphA1*-IS*26* and *floR*-IS*CR2*-IS*26*. It harbors an atypical class 1 integron (5′CS-*sat*-*psp*-*aadA2*-*cmlA1*-*aadA1*-*qacH*-IS*440*-*sul3*) belonging to *sul3*-type III (Antunes et al., 2007). The class 1 integron adjacent to the *res* gene (S027) carries gene cassettes *dfrA1*-*orfC*. This region contains five copies of IS*26* in the same orientation. However, the 8-bp target site duplications could not be observed around these IS*26*, suggesting this complicated MDR region might be formed via the incorporation of several IS*26*-mediated translocatable units successively (Harmer and Hall, 2015, 2016). The right end of SGI1-PmSC1111 harbors four gene cassettes, *aac(6*′*)-Ib-cr* (fluoroquinolones and aminoglycosides), *bla*_OXA–__1_ (ampicillin), *catB3* (chloramphenicol) and *arr-3* (rifampicin) never reported to date in SGI1.

### Mobilization of SGI1

A free circle can be formed after excision of SGI1 from the chromosome (Doublet et al., 2005). SGI1 appeared to be non-self-transmissible, but it could potentially be integrated into the chromosome of another bacterial species by the help of IncA/C plasmid (Douard et al., 2010; Siebor et al., 2016). In the recipient strain, the circular form of SGI1 integrates in a specific site at the 3′ end of the chromosomal *trmE* gene (Doublet et al., 2005).

The circular forms of SGI1 in all strains except for PmBC1123 and PmSC1111 were detected by two rounds of PCR amplification. We did not detect the circular form of SGI1-PmBC1123 because of the inversion of the right direct repeat in SGI1-PmBC1123. The circular form of SGI1-PmSC1111 could not be detected through three independent experiments. Mobilization assays showed that the three known SGI1 variants (SGI1-F, SGI1-I and SGI1-W) in *S. enterica* or *P. mirabilis* could be conjugally mobilized to *E. coli* and was incorporated into the 3′-end of *trmE*. The conjugative transfer of them were detected at frequencies between 10^–6^ and 10^–7^, suggesting that these SGI1s can be transferred between bacterial species (Table 2). However, the mobilization of SGI1-PmBC1123 and SGI1-PmSC1111 failed despite three independent attempts. The results indicate that the sequence inversion of partial SGI1 backbone may result in the loss of mobility of SGI1. We supposed that the inversion of the right direct repeat in SGI1-PmBC1123 may lead to losing the capability to form a circular form and then mobility. Nevertheless, in SGI1-PmSC1111, IS*26*-mediated rearrangements resulted in inversions of the backbone was supposed to affect the expression of some genes related to mobilization. The mechanism needs further study to clarify.

**TABLE 2:** Conjugative transfer frequency of *S. enterica* or *P. mirabilis* SGI1s.

**Donor strain**	**SGI1 variant**	**Conjugative plasmid**	**SGI1 transfer frequency**
SCMYP1	SGI1-F	IncC plasmid pR55	1.7 × 10^–6^
SC10	SGI1-F	IncC plasmid pR55	1.9 × 10^–6^
SC968	SGI1-I	IncC plasmid pR55	2.3 × 10^–7^
PmBC55	SGI1-W	IncC plasmid pR55	4.3 × 10^–6^
PmSN55	SGI1-W	IncC plasmid pR55	3.7 × 10^–6^
PmDJ107	SGI1-W	IncC plasmid pR55	3.4 × 10^–6^

### Other Resistance Genes That Were Not Associated With SGI1

The acquired antimicrobial resistance genes in SGI1-containing *S. enterica* and *P. mirabilis* isolates are listed in Table 3. *S*. Albany strain SCMYP1 harbors *bla*_CTX–M–__55_ and *mcr-1*, explaining the resistance to third generation cephalosporins and polymyxin B, respectively. *P. mirabilis* strains PmSN55 and PmDJ107 harbors AmpC cephalosporinase gene *bla*_CMY–__2_ carried by an 11.7-kb contig that is identical to the corresponding region of SXT/R391 ICE ICE*Pmi*Jpn1 (Harada et al., 2010; Lei et al., 2016), indicating the *bla*_CMY–__2_ gene in those two strains might be carried by ICE*Pmi*Jpn1. It is notable that strain PmSC1111 harbors the multiresistance gene *cfr* that is also carried by SXT/R391 ICE (accession no. CP034090). Very recently we reported a novel SXT/R391 ICE that carried *cfr*, *bla*_CTX–M–__65_, *fosA3*, and *aac(6*′*)-Ib-cr* in *P. mirabilis* and could be transferred to *E. coli* (Lei et al., 2018a). Taken together, SXT/R391 ICE could mediate the dissemination of clinically important resistance genes in *P. mirabilis*, which needs to draw more attention.

**TABLE 3:** Acquired antimicrobial resistance genes in SGI1-containing *S. enterica* and *P. mirabilis* isolates.

**Strain**	**Aminoglycoside *^a^***	**β-lactam**	**Colistin**	**Fluoroquinolone**	**MLS – Macrolide, Lincosamide and Streptogramin B**	**Phenicol**	**Rifampicin**	**Sulfonamide**	**Tetracycline**	**Trimethoprim**
SCMYP1		***bla*_CARB–__2_** *bla*_CTX–M–__55_	*mcr-1*			***floR***		***sul1***	***tetA*(G)**	***dfrA1***
SC10		***bla*_CARB–__2_**				***floR***		***sul1***	***tetA*(G)**	***dfrA1***
SC968	***aadA2***					***floR***		***sul1***	***tetA*(G)**	***dfrA1***
PmBC55	*strB strA hph aacC4 aadA1* ***aadA2***				***lnu(F)***	*floR catB2*		***sul1*** *sul2*	*tetA*(A)	*dfrA12 dfrA1*
PmBC1123	*aphA1 strA strB* ***aacC4 hph aadA2***	*bla*_OXA–__1_		*aac(6*′*)-Ib-cr*	*ere(A)* ***lnu(F)***	*catB4* ***floR***	*arr-3*	***sul1 sul2***	*tetA*(C)	***dfrA12*** *dfrA32*
PmSC1111	***hph aacC4 aphA1*** *strA strB aadA1* ***aadA2***	***bla*_OXA–__1_**		***aac(6***′***)-Ib-cr***	*ere(A) cfr mph(E) msr(E)*	***floR cmlA1 catB3***	***arr-3***	***sul1 sul2 sul3***	*tetA*(C) *tetA*(D)	***dfrA12*** *dfrA32 dfrA1*
PmSN55	*hph aacC4 aphA1* ***aadA2*** *aadA14*	*bla*_CMY–__2_			***lnu(F)***	*floR*		***sul1*** *sul2*		*dfrA12*
PmDJ107	*hph aacC4 aphA1 aadA1* ***aadA2***	*bla*_CMY–__2_			***lnu(F)***	*floR*		***sul1*** *sul2*		*dfrA12 dfrA1*

*^*a*^Antimicrobial resistance genes carried by SGI1 are indicated in bold. *tetA(J)* is inherent in the chromosome. ^*b*^Acquired antimicrobial resistance genes were identified by ResFinder 3.1 (https://cge.cbs.dtu.dk/services/ResFinder/).*

## Conclusion

In this study, we characterized SGI1 in *S. enterica* and *P. mirabilis* of food-producing animal origin in Sichuan Province and described two novel SGI1 variants, SGI1-PmBC1123 and SGI1-PmSC1111. The *sul3*-type III class 1 integron (5′CS-*sat*-*psp*-*aadA2*-*cmlA1*-*aadA1*-*qacH*-IS440-*sul3*) and gene cassettes *aac(6*′*)-Ib-cr*-*bla*_OXA–__1_-*catB3*-*arr-3* are reported in SGI1 for the first time. Our study highlights that IS*26*-mediated rearrangements promote the diversity of SGI1.

## Data Availability Statement

The datasets generated for this study can be found in the GeneBank, MH998664, MH998665, CP034091 and CP034090.

## Ethics Statement

This study was carried out in accordance with the recommendation of ethical guidelines of Sichuan University. The protocol was approved by the Sichuan University Animal Ethics Committee. Individual informed consent for the use of samples was obtained from all the animal owners.

## Author Contributions

X-CW, C-WL, Z-ZK, and YZ performed the experiments. C-WL analyzed the data and conceived of the study. X-CW, C-WL, and Z-ZK wrote the manuscript. All authors contributed to manuscript revision and approved the final manuscript.

## Conflict of Interest

The authors declare that the research was conducted in the absence of any commercial or financial relationships that could be construed as a potential conflict of interest.

## Footnotes

1 https://cge.cbs.dtu.dk/services/MLST/

2 https://cge.cbs.dtu.dk/services/ResFinder/

3 http://blast.ncbi.nlm.nih.gov/Blast.cgi

4 https://cge.cbs.dtu.dk/services/CSIPhylogeny/
