# Supplementary material for: IS26-Mediated Genetic Rearrangements in Salmonella Genomic Island 1 of Proteus mirabilis
Source: Front Microbiol. 2019 Sep 24;10:2245. doi: 10.3389/fmicb.2019.02245 (PMC6769106; doi:10.3389/fmicb.2019.02245)
Supplement: Supplementary file 1 [file Table_1.DOCX]

Supplementary Material

**Table S1.** SGI1 and SGI1 relative genomic island in *P. mirabilis*

| GI variants | Size (bp) *^b^* | Source | Country | Year | Resistance genes *^c^* | Genebank accession No. | Reference |
| --- | --- | --- | --- | --- | --- | --- | --- |
| SGI1 | - | Human | China | - | *aadA2*, *floR*, *tetA*(G), *bla*_CARB-2_, *sul1* | - | Boyd et al., 2008 |
|  | 42,432 | Chicken | China | 2012 | *aadA2*, *floR*, *tetA*(G), *bla*_CARB-2_, *sul1* | KJ186153 | Lei et al., 2014 |
| SGI1-A | - | Dog | France | 2011 | *aadA2*, *floR*, *tetA*(G), *bla*_CARB-2_, *sul1*, *dfrA10* | - | Schultz et al., 2015 |
| SGI1-B | 33,159 | Human | China | 2014 | *bla*_CARB-2_, *sul1* | MH990675 | Xiao et al., 2019 |
|  | 33,159 | Human | China | 2015 | *bla*_CARB-2_, *sul1* | MH990682 | Xiao et al., 2019 |
|  | 33,160 | Human | China | 2012 | *bla*_CARB-2_, *sul1* | KU987430 | - |
| SGI1-B2 | 45,929 | Swine | China | 2013 | *dfrA17*, *aadA5*, *sul1*, *mphR*, *mrx*, *mphA, bla*_CARB-2_ | KP116299 | Lei et al., 2015 |
| SGI1-C | 32,972 | Human | China | 2014 | *aadA2*, *sul1* | MH990676 | Xiao et al., 2019 |
|  | 32,972 | Human | China | 2015 | *aadA2*, *sul1* | MH990679 | Xiao et al., 2019 |
| SGI1-H | - | Dog | France | 2010 | *aadA7*, *floR*, *tetA*(G), *bla*_CARB-2_, *sul1* | - | Schultz et al., 2015 |
|  | - | Dog | France | 2015 | *aadA7*, *floR*, *tetA*(G), *bla*_CARB-2_, *sul1* | - | Schultz et al., 2017 |
| SGI1-I | - | Food | China | - | *aadA2*, *floR*, *tetA*(G), *dfrA1*, *sul1* | - | Boyd et al., 2008 |
|  | 42,477 | Chicken | China | 2012 | *aadA2*, *floR*, *tet*(G), *dfrA1*, *sul1* | KJ186152 | Lei et al., 2014 |
|  | 42,477 | Food | China | 2009 | *aadA2*, *floR*, *tet*(G), *dfrA1*, *sul1* | MH990671 | Xiao et al., 2019 |
| SGI1-K7 | 55,143 | Human | France | 2014 | *strA*, *strB*, *tetA*(A), *sul1*, *aadA7*, *aacCA5*, *bla*_TEM-1b_, *aacC2*, *tmrB*, *bla*_CTX-M-15_ | MF372717 | Curraize et al., 2018 |
| SGI1-L | - | Human | Palestine | 2006 | *dfrA15*, *floR*, *tetA*(G), *bla*_CARB-2_, *sul1* | - | Ahmed et al., 2007 |
|  | - | Dog | France | 2013 | *dfrA15*, *floR*, *tetA*(G), *bla*_CARB-2_, *sul1* | - | Schultz et al., 2015 |
| SGI1-O | - | Human | China | - | *dfrA1*, *sul1* | - | Boyd et al., 2008 |
|  | - | Food | China | - | *dfrA1*, *sul1* | - | Boyd et al.,2008 |
|  | - | Human | France | 2007 | *dfrA1*, *sul1* | - | Doublet et al., 2010 |
|  | 33,205 | Chicken | China | 2012 | *dfrA1*, *sul1* | KJ186150 | Lei et al., 2014 |
|  | 33,206 | Food | China | 2008 | *dfrA1*, *sul1* | MH990670 | Xiao et al., 2019 |
|  | 33,205 | Human | China | 2015 | *dfrA1*, *sul1* | MH990677 | Xiao et al., 2019 |
|  | 33,205 | Human | China | 2013 | *dfrA1*, *sul1* | KU987431 | - |
| SGI1-U | - | Human | China | 2006-2008 | *dfrA5*, *sul1* | - | Bi et al., 2011 |
|  | - | Chicken meat | China | 2009 | *dfrA5*, *sul1* | - | Bi et al., 2011 |
| SGI1-V | 42,904 | Human | France | 2009 | *aacA4*, *aadB*, *dfrA1*, *sul1*, *tetA*(A), *bla*_VEB-6_, *qnrA1* | HQ888851 | Siebor et al., 2011 |
|  | - | Dog | France | 2013 | *aacA4*, *aadB*, *dfrA1*, *sul1*, *tetA*(A), *bla*_VEB-6_, *qnrA1* | - | Schultz et al., 2015 |
|  | - | Human | France | 2013 | *aacA4*, *aadB*, *dfrA1*, *sul1*, *tetA*(A), *bla*_VEB-6_, *qnrA1* | - | Schultz et al., 2015 |
|  | - | Dog | France | 2013-2014 | *aacA4*, *aadB*, *dfrA1*, *sul1*, *tetA*(A), *bla*_VEB-6_, *qnrA1* | - | Schultz et al., 2017 |
|  | - | Horse | France | 2013 | *aacA4*, *aadB*, *dfrA1*, *sul1*, *tetA*(A), *bla*_VEB-6_, *qnrA1,* | - | Schultz et al., 2017 |
| SGI1-W | 33,909 | Chicken | China | 2012 | *aadA2*, *lnuF*, *sul1* | KJ186151 | Lei et al., 2014 |
|  | - | Human | Egypt | 2015 | *aadA2*, *lnuF*, *sul1* | - | Soliman et al., 2017 |
|  | - | Chicken | Egypt | 2016 | *aadA2*, *lnuF*, *sul1* | - | Soliman et al., 2018 |
| SGI1-X | 38,405 | Chicken | China | 2012 | *dfrA25*, *qnrB2*, *sul1* | KJ186154 | Lei et al., 2014 |
| SGI1-Y | 33,542 | Chicken | China | 2012 | *aacCA5*, *aadA7*, *sul1* | KJ186149 | Lei et al., 2014 |
| SGI1-Z | 34,126 | Human | China | 2013 | *dfrA12*, *aadA2*, *sul1* | KP662516 | Qin et al., 2015 |
|  | 33,876 | Human | China | 2015 | *dfrA12*, *aadA2*, *sul1* | MH990681 | Xiao et al., 2019 |
| SGI1-B0610 | 32,723 | Human | China | 2012 | *aadB*, *sul1* | KU987432 | - |
| SGI1-*Pm*ABB | 32,021 | Human | France | 2012 | *aacCA5*, *aadA7*, *sul1* | JX121638 | Siebor et al., 2013 |
|  | 32,021 | Swine | China | 2014 | *aacCA5*, *aadA7*, *sul1* | KP313760 | Lei et al., 2015 |
|  | - | Human | France | 2012 | *aacCA5*, *aadA7*, *sul1* | - | Schultz et al., 2015 |
|  | - | Dog | France | 2014 | *aacCA5*, *aadA7*, *sul1* | - | Schultz et al., 2017 |
|  | - | Human | Egypt | 2014 | *aacCA5*, *aadA7*, *sul1* | - | Soliman et al., 2017 |
|  | - | Chicken | Korea | 2013-2014 | *aacCA5*, *aadA7*, *sul1* | - | Sung et al., 2017 |
|  | - | Chicken | China | 2013 | *aacCA5*, *aadA7*, *sul1* | - | Bie et al., 2018 |
| SGI1-*Pm*BRI | 32,972 | Human | France | 2011 | *aadA2*, *sul1* | JX089582 | Siebor et al., 2013 |
| SGI1-*Pm*CAU | 33,205 | Human | France | 2009 | *dfrA1*, *sul1* | JX089581 | Siebor et al., 2013 |
|  | - | Chicken | China | 2013 | *dfrA1*, *sul1* | - | Bie et al., 2018 |
| SGI1-*Pm*CA11 | 35,131 | Human | China | 2012 | *dfrA14*, *arr-2*, *bla*_OXA-10_, *aadA15*, *sul1* | MH990673 | Xiao et al., 2019 |
| SGI1-*Pm*CA14 | 43,469 | Human | China | 2012 | *aadB*, *cmlA5*, *sul1* | MH990672 | Xiao et al., 2019 |
| SGI1-*Pm*CA46 | 37,849 | Human | China | 2014 | *bla*_CARB-2_, *aacA4*, *bla*_OXA-1,_ *catB3*, *arr-3*, *dfrA17*, *aadA5*, *sul1* | MH990674 | Xiao et al., 2019 |
| SGI1-*Pm*GUE | 66,110 | Human | France | 2012 | *aacCA5*, *aadA7*, *tetA*(A), *strB*, *strA*, *bla*_TEM-1b_, *aphA1a*, *dfrA15*, *floR*, *tetA*(G), *bla*_CARB-2_, *sul1* | JX121641 | Siebor et al., 2013 |
| SGI1-*Pm*JN16 | 34,961 | Chicken | China | 2013 | *dfrA12*, *aad2*, *sul1* | MF576129 | Bie et al., 2018 |
| SGI1-*Pm*JN40 | 56,713 | Chicken | China | 2013 | *aadA2*, *sul1* | MF576128 | Bie et al., 2018 |
| SGI1-*Pm*JN48 | 189,052 | Chicken | China | 2013 | *bla*_CARB-2_, *sul1*, *mphR*, *mrx*, *mphA*, *arr3*, *cat3*, *bla*_OXA−1_, *aac(6')-Ib-cr*, *bla*_CTX−M-65_, *dfrA17*, *aadA5* | MF576130 | Bie et al., 2018 |
| SGI1-*Pm*MAT | 30,760 | Human | France | 2011 | *aacCA5*, *aadA7*, *sul1* | JX089583 | Siebor et al., 2013 |
|  | - | Dog | France | 2014 | *aacCA5*, *aadA7*, *sul1* | - | Schultz et al., 2017 |
| SGI1-*Pm*SCO | 41,481 | Human | France | 2011 | *aacCA5*, *aadA7*, *floR*, *tetA*(G), *bla*_CARB-2_, *sul1* | JX121639 | Siebor et al., 2013 |
| SGI1-*Pm*SC42 *^a^* | 24,297 | Swine | China | 2013 | None | KP057606 | Lei et al., 2015 |
| SGI1-*Pm*VER | 53,323 | Human | France | 2010 | *aacCA5*, *aadA7*, *tetA*(A), *strB*, *strA*, *bla*_TEM-1b_, *aphA1a*, *sul1* | JX121640 | Siebor et al., 2013 |
| SGI1-*Pm*2CHAMA | 53,552 | Human | France | 2013 | *qacH*, *aadA1*, *aphA1a*, *strA*, *strB*, *bla*_CARB-4_, *dfrA15*, *sul1* | MF372716 | Curraize et al., 2018 |
| SGI1-*Pm*SC1111 | 82,352 | Swine | China | 2016 | *aacC4*, *hph*, *sul2*, *floR*, *sat*, *aadA2*, *cmlA1*, *qacH*, *sul3*, *aphA1*, *dfrA12*, *sul1*, *dfrA1*, *aac(6’)-1b-cr*, *bla*_OXA-1_, *catB3*, *arr-3* | MH998665 | in this study |
| SGI1-*Pm*BC1123 | 58,069 | Swine | China | 2016 | *dfrA12*, *aadA2*, *sul1*, *lnuF*, *floR*, *sul2*, *hph*, *aacC4* | MH998664 | in this study |
|  |  |  |  |  |  |  |  |
| PGI1-*Pm*CHA | 81,146 | Human | France | 2012 | *aadB*, *aadA2*, *sul1*, *bla*_TEM-135_, *aphA1b*, *aadA1*, *tetA*(A), *strB*, *strA* | KJ411925 | Siebor et al., 2014 |
| PGI1-*Pm*CHE | 78, 972 | Human | France | 2012 | *aadB*, *aadA2*, *sul1*, *bla*_TEM-135_, *aadA1*, *tetA*(A), *strB*, *strA* | KJ439039 | Siebor et al., 2014 |
| PGI1-*Pm*ESC | 42,436 | Human | France | - | *aadB*, *aadA2*, *sul1* | KU499917 | Siebor et al., 2016 |
| PGI1-*Pm*PEL | 64,402 | Human | France | 2012 | *aacA4*, *aadB*, *dfrA1*, *sul1*, *tetA*(A), *bla*_VEB-6_, *aphA6*, *bla*_NDM-1_, *bla*_DHA-1_ | KF856624 | Girlich et al., 2015 |
| PGI2 | 61,578 | Swine | China | 2016 | *dfrA16*, *bla*_CARB-2_, *aadA2*, *cmlA1*, *aadA1*, *sul1*, *blms*, *aphA1*, *aacC4*, *hph*, *sul2*, *floR*, *aadA2*, *lnuF* | MG201402 | Lei et al., 2018 |
| PGI2-*Pm*CA72 | 42,797 | Human | China | 2015 | *dfrA16*, *bla*_CARB-2_, *aadA2*, *cmlA1*, *aadA1*, *sul1*, *floR*, *tetA*(A) | MH990678 | Xiao et al., 2019 |
| GI*Pmi*1 | 55,853 | Human | France | 2012 | *qacE*, *strB*, *strA*, *bla*_TEM-1b_, *sul1*, *aadA2*, *aadB* | MF490433 | Siebor et al., 2018 |

***^a^*** SGI1-*Pm*SC42, formerly named SGI1-Z.

*^b^* The size of complete genomic island is an estimation as the available GenBank entry.

*^c^* *tmrB*, tunicamycin resistance gene;*blms*, bleomycin resistance gene; *hph*, hygromycin B phosphotransferase gene.

**Table S2.** Primers used in this study

| Primers | Nucleotide sequence (5’-3’) | Reference |
| --- | --- | --- |
| Detection of SGI1-relative GIs | |  |
| FwintSGI1HR / RvintSGI1HR | ATGTTGCGTCAGGCYGAGGC / GAGTGYCCAAGAAGSCGAGAG | (Schultz et al., 2017) |
| int-AGI-F /int-AGI-R | CACGAGCCACTAGACATC/ TGAAGTAGCGCTACGTTG | (Siebor et al., 2019) |
| GIP*mi*1-F /GIP*mi*1-R | CTCGGAAGACGGCTCAAGA/ AGTACATCGTAACGGCATTCTG | in this study |
| Detection of circular extrachromosomal form of SGI1-*Pm*SC1111 | | |
| SC1111-F1 /SC1111-R1 | ATCATCCCGACGACAATACAGA/ CCATCCTCACCTTCAACAACTC | in this study |
| SC1111-F2 /SC1111-R2 | TACGATCTGGTAGGCAGTGAC/ CGAGTTAGGGTTACGCTTGTTT |  |
| Detection of transconjugants | |  |
| trmE-Ecoli-mF / LJ-R1 | CCGCGTGCTGTTTATGGT/ AGTTCTAAAGGTTCGTAGTCG | (Boyd et al., 2001; Siebor et al., 2016) |
| 104-RJ / tnaA-Ecoli-outR | TGACGAGCTGAAGCGAATTG/ CTGTCGGTCAGTAAATCG |  |

**References**

Boyd, D., Peters, G.A., Cloeckaert, A., Boumedine, K.S., Chaslus-Dancla, E., Imberechts, H., et al. (2001). Complete nucleotide sequence of a 43-kilobase genomic island associated with the multidrug resistance region of Salmonella enterica serovar Typhimurium DT104 and its identification in phage type DT120 and serovar Agona. *J Bacteriol* 183(19)**,** 5725-5732. doi: 10.1128/jb.183.19.5725-5732.2001.

Schultz, E., Cloeckaert, A., Doublet, B., Madec, J.Y., and Haenni, M. (2017). Detection of SGI1/PGI1 Elements and Resistance to Extended-Spectrum Cephalosporins in Proteae of Animal Origin in France. *Front Microbiol* 8**,** 32. doi: 10.3389/fmicb.2017.00032.

Siebor, E., de Curraize, C., Amoureux, L., and Neuwirth, C. (2016). Mobilization of the Salmonella genomic island SGI1 and the Proteus genomic island PGI1 by the A/C2 plasmid carrying blaTEM-24 harboured by various clinical species of Enterobacteriaceae. *J Antimicrob Chemother* 71(8)**,** 2167-2170. doi: 10.1093/jac/dkw151.

Siebor, E., de Curraize, C., and Neuwirth, C. (2019). Identification of AGI1-A, a variant of Acinetobacter genomic island 1 (AGI1), in a French clinical isolate belonging to the Enterobacter cloacae complex. *J Antimicrob Chemother* 74(2)**,** 311-314. doi: 10.1093/jac/dky442.
